# The American and Colonial Journals: Medicine

**Published:** 1839-04

**Authors:** 


### II. THE AMERICAN AND COLONIAL JOURNALS.

### MEDICINE.

*Remarks on Pneumothorax, with cases, and an experimental enquiry into the causes of the metallic sounds heard in that disease.* By JACOB BIGELOW, M.D., Professor of Materia Medica and of Clinical Medicine in Harvard University.

[THIS paper contains the detail of three fatal cases of pneumothorax, and of some experiments made on the bodies after death, with the view to ascertain the causes of the sounds observed in this disease. We have only room for the conclusions arrived at.]

From the foregoing experiments and cases (says Dr. Bigelow), we may infer that the following agencies are concerned in producing metallic sounds of the chest.

1. There must be a cavity, the walls of which are preternaturally susceptible of vibration. This takes place when the pleura is pathologically distended, so as to overcome the obtuse or muffling effect of the contiguous soft organs, such as the lung, diaphragm, and intercostal muscles. Some time is probably necessary to prepare the parts for this pathological resonance, since it fails to appear *post mortem* in healthy chests submitted to experiment. It should be added that when metallic sounds appear in simple phthisis, there are cavities of the lungs, the walls of which are in a state of tubercular induration.

2. The immediate or exciting cause of metallic tinkling, is a forcible or sudden disturbance of the liquid in a vibrating cavity like that described. The explosion of bubbles of air from beneath the surface of the liquid appears to be the most common cause of such a disturbance; but it may also take place when a part of the liquid is thrown upward in the act of coughing, and falls back upon the remainder. The same occurs in succussion of the chest.

3. The vibrations which yield metallic tinkling are transmitted from the liquid to the solid parietes, and thence directly to the ear, without any necessary agency of an echo, or reverberation of air in the cavity.

4. A minor or *submetallic* tinkling, having no musical resonance, may be produced by slight impulses given to the air in the cavity, such as the breaking of bubbles of mucus at orifices above the surface of the liquid.

5. Amphoric resonance is produced by reverberations of the air in a vibrating cavity, without sonific impulse of the liquid. The same is true of metallic modifications of the voice, and of the cough when there is no tinkling. Metallic percussion seems also to depend upon the vibrations of air independently of liquid, and may be produced in some other cases when we strike upon a tense cavity in which a certain quantity of air is confined.

*American Journal of Med. Sciences. Nov.* 1838.

